# Compatibility Between Physical Stimulus Size – Spatial Position and False Recognitions

**DOI:** 10.3389/fpsyg.2018.01457

**Published:** 2018-08-14

**Authors:** Seda Dural, Birce B. Burhanoǧlu, Nilsu Ekinci, Emre Gürbüz, İdil U. Akın, Seda Can, Hakan Çetinkaya

**Affiliations:** ^1^Department of Psychology, İzmir University of Economics, İzmir, Turkey; ^2^Department of Psychology, Ankara University, Ankara, Turkey

**Keywords:** size–space compatibility, object size, false memory, signal detection, accuracy of recall, reaction time, recall bias

## Abstract

Magnitude processing is of great interest to researchers because it requires integration of quantity related information in memory regardless of whether the focus is numerical or non-numerical magnitudes. The previous work has suggested an interplay between pre-existing semantic information about number–space relationship in processes of encoding and recall. Investigation of the compatibility between physical stimulus size – spatial position and false recognition may provide valuable information about the cognitive representation of non-numerical magnitudes. Therefore, we applied a false memory procedure to a series of non-numerical stimulus pairs. Three versions of the pairs were used: big-right (a big character on the right/a small character on the left), big-left (a big character on the left/a small character on the right), and equal-sized (an equal sized character on each side). In the first phase, participants (*N* = 100) received 27 pairs, with nine pairs from each experimental condition. In the second phase, nine pairs from each of three stimulus categories were presented: (1) original pairs that were presented in the first phase, (2) mirrored pairs that were horizontally flipped versions of the pairs presented in the first phase, and (3) novel pairs that had not been presented before. The participants were instructed to press “YES” for the pairs that they remembered seeing before and to press “NO” for the pairs that they did not remember from the first phase. The results indicated that the participants made more false-alarm responses by responding “yes” to the pairs with the bigger one on the right. Moreover, they responded to the previously seen figures with the big one on the right faster compared to their distracting counterparts. The study provided evidence for the relationship between stimulus physical size and how they processed spatially by employing a false memory procedure. We offered a size–space compatibility account based on the congruency between the short- and long-term associations which produce local compatibilities. Accordingly, the compatible stimuli in the learning phase might be responsible for the interference, reflecting a possible short-term interference effect on congruency between the short- and long-term associations. Clearly, future research is required to test this speculative position.

## Introduction

The last two decades have witnessed a flurry of research activity regarding the understanding of extent and nature of number–space association. This activity has been influenced, at least in part, by the work of Dehaene et al. (1990, 1993). They demonstrated that left-hand responses were faster to small as compared to large numbers, whereas the reverse was true for right-hand responses. Moreover, this number–space (response side) compatibility effect was also evident in tasks such as parity judgment that did not require encoding the magnitude of the numbers given. These findings were found to be in line with the metaphor of mental number line (MNL). According to the MNL, numerical representations of magnitudes tend to be spatially organized and the representation of numerical information takes place on an ascending left-to-right oriented line. Based on this, Dehaene et al. (1993) proposed so-called the spatial-numerical association of response codes (SNARC) effect and used the concept of MNL as an account for the SNARC. Since its introduction, several studies have challenged implications of SNARC. Hence, the subsequent research showed that even though the spatial organization of cognitive representations of quantities might be adaptive, the direction and strength of the effect are neither automatic nor unchanging anchors, but are flexible (e.g., Fischer, 2006; Lindemann et al., 2008; Santens and Gevers, 2008; Fischer and Shaki, 2014; Ginsburg et al., 2014; Ginsburg and Gevers, 2015). In addition, according to Proctor and Cho (2006) the endpoints of conceptual dimensions (e.g., tall-short, happy-sad, big-small, etc.) do not share the same representational status, they differ in their valences. People tend to code the stimulus and response alternatives as + polarity and – polarity. Hence, the polarity correspondence account (Proctor and Cho, 2006) predicts that the response selection is faster when the polarities correspond than when they do not. The approach further predicts that the valence of a given pole may be experimentally reversed, because they are largely defined by the relevant context (as in Banks et al., 1975). Although the SNARC effect was largely attributed to representing the numbers along a horizontal line, it may be a consequence of coding large as + polarity and small as – polarity. Therefore, MNL may have originated from ontogenetically acquired behaviors, such as counting (Opfer et al., 2010; Shaki et al., 2012a) or reading and writing habits (Dehaene et al., 1993). As most languages around the world share left-to-right reading and writing direction, MNL appears to be a largely culture-specific, developmentally shaped representational tool which enables efficient coding and comparison of the meaning of magnitudes (Tzelgov et al., 1992).

Magnitude processing is of great interest to researchers because it guides action by integrating information about temporal, spatial, and quantity aspects of the action. Given its significance in survival, the neural mechanism of magnitude processing probably originated from a shared evolutionary history (Hubbard et al., 2005; Cantlon et al., 2009), and thus it might be reasonable to conceptualize a generalized magnitude processing system. In fact, a prominent generalized theory of magnitude (ATOM) (Walsh, 2003, 2015) has already been formulated. According to the theory, information about time, space, and quantity likely share a common spatial processing mechanism in the brain, due to similarities in their mapping metrics. In line with the theory, the growing body of empirical evidence suggests that a generalized core system may be responsible for the processing of magnitude of different dimensions. The evidence from behavioral studies revealed the relationship between various dimensions, including time, size, letters, luminance (see for reviews Winter et al., 2015; Macnamara et al., 2018), and neurobiological works showed overlapping neural circuits in human parietal cortex for the representation of number, size, and luminance (e.g., Pinel et al., 2001; Fias et al., 2003; Cohen Kadosh et al., 2007; Bueti and Walsh, 2009; Skagerlund et al., 2016).

Although the ATOM hypothesized a general magnitude code serving across diverse quantifiable dimensions, curiously, there has been little work on the relationship between physical size and response location (for a concise review, see Wühr and Seegelke, 2018). Compared to other domains (e.g., number–space and number–size), very few studies (e.g., Ren et al., 2011; Sellaro et al., 2015; Dural et al., 2017; Wühr and Seegelke, 2018) addressed the size and space interaction. For example, in a typical magnitude comparison task, Wühr and Seegelke (2018) found a significant stimulus size–response side compatibility effect when participants were instructed to press left key for the small square, and right key for the large square presented at the center of screen. Participants responded faster to the smaller figure with left key, and faster to the larger figure with right key. They were able to replicate the findings when participants responded to seemingly irrelevant feature (color) of small and large squares. Similarly, Sellaro et al. (2015) asked participants to decide whether the target stimulus was larger or smaller than a reference stimulus with their either right or left index finger. Results revealed a SNARC-like effect: Compared to a reference object, smaller objects were associated with shorter left-side reaction times, and larger objects were associated with shorter right-side reaction times (see Ren et al., 2011; Shaki et al., 2012b, for similar findings). Rather than measuring reaction times, Dural et al. (2017) focused on imagery codes. They presented participants pairs of words referring to objects of varying size differences (e.g., high difference: mouse – elephant, low difference: horse – zebra, average difference: microwave – toaster) and asked to visualize the objects as clearly as possible with eyes closed. After opening their eyes, the participants were instructed to indicate with either left or right hand the location of the imagined objects on the screen divided into halves by a vertical line (e.g., mouse on the left, elephant on the right). Findings showed the tendency to visualize the bigger object on the right increased proportionally with the size difference between the two stimuli, and visualizations of objects seemed to follow an ascending size order from left to right, independent of the hand used to indicate the side of their imagined object. In line with the polarity hypothesis (Proctor and Cho, 2006), the effect tended to diminish as the size difference between the imagined object pairs decreased. These studies provide evidence for a link between mental representations of physical size and space, and this suggestive link manifests itself not only in participants’ faster motor responses for the compatible physical stimulus size and left-right response conditions, but also in how they locate stimuli in space based on relative size. Thus, conceivably, long-term representations play role in physical size and response-side interactions.

In line with the ATOM, successful regulation of action requires integration of quantity related information in memory regardless of whether the focus is numerical or non-numerical magnitudes. The previous work has indicated an interplay between pre-existing semantic information about number–space relationship in processes of encoding and recall. For example, arbitrarily ordered numerical information is not readily stored in the long-term memory; and so, it requires extra effort for acquisition (i.e., training for learning). This working memory account (van Dijck and Fias, 2011) implies that ordinal information is spatially organized not only in long-term memory (Zhang et al., 2016), but also in working memory (Lindemann et al., 2008; Fias et al., 2011; van Dijck and Fias, 2011; Ginsburg and Gevers, 2015). Although recent works have shed light on the role of STM on number–space relationship, to the best of our knowledge, there is only one study (Gut and Staniszewski, 2016) that explicitly addressed the effects of interaction between STM and LTM on the number–space relationship. More specifically, their focus was on how the relatively solid MNL representation modulates the recall of numerical information from STM, regarding the number magnitude–response side congruency. The task they employed required participants to retrieve the spatial position of a digit displayed in the row of four digits which were varied in magnitude. They found that the memorization and retrieval of numbers from STM was more effective when numbers are presented congruently with their position on the LTM.

In cases in which a false memory occurs, participants wrongfully attribute pre-existing semantic information to an external source (Johnson et al., 1993). Thus, memory errors, especially the false alarms, and reaction time measures in recognition may provide helpful data in the understanding of cognitive mechanisms of spatial representations of magnitudes. However, there is so far no evidence of memory influences on relationship between physical size and spatial location of responding and on recall latency and accuracy. If a generalized magnitude coding system is in charge of processing spatially sensitive magnitude information, then it should be possible to identify similar physical size effects on memory performance (e.g., recognition memory) as on numerical magnitude.

In present study, we aimed to investigate the effects of congruency between short- and long-term associations on encoding and retrieval processes in a SNARC-like size–space compatibility by employing a false memory procedure. We manipulated two variables as experimental condition (big-right, big-left, and equal-sized), and stimulus category (original, mirrored, and novel). In the first variable, the big-right and the big-left represented the compatibility and incompatibility conditions, respectively. The equal-sized served as a control condition. In the second variable, as a part of false memory procedure, the original referred to a previously shown stimulus, and the mirrored and novel served as distractors. The study consisted of two main phases as learning and test phases. In the learning phase, we presented a series of non-numerical, arbitrary pairs of figures, which varied in terms of their relative physical size and spatial position (big-right, big-left, and equal-sized). In the test phase, the participants were tested by original (the same pairs as in learning phase), mirrored (the mirrored versions of the same pairs shown in learning phase), and novel stimulus pairs (the ones never shown in learning phase). The pairs in both learning and test phases always contained identical types of characters. The participants were instructed to indicate as accurately and quickly as possible whether each pair of figures had been seen in a previous phase (i.e., learning phase) of the study. Therefore, the task required comparing the available information (pairs of figures to be tested) with some internal criteria (spatial magnitude representations) that provide guidance on recognition. We evaluated how accurately and how fast participants performed the task.

As may be the case for the numerical comparison tasks, our main prediction is that interaction between memory-dependent information regarding stimulus size and position may interact to elicit a SNARC-like effect. In order to test this, the accuracy and reaction time measures were taken into consideration. We applied Signal Detection Theory to determine discrimination index (*d*′) and response bias (*c*) based on the observed recognition accuracy in different test conditions. We hypothesize that in comparisons of the previously seen stimulus (original) and the distractors (mirrored and novel), there will be smaller *d*′ values, indicating that participants cannot discriminate signals (previously seen stimuli) from the noise (distractor) when there is compatibility between size and space (i.e., big-right condition). We also expect that the participants will have negative *c* values in compatible condition, showing a tendency to favor “yes” responses. That is, the semantic map of physical size, which presumably resides in long-term memory, will lead more false recognitions. We also make predictions about the reaction time measures as follows: For the original stimuli, we predict shorter reaction times to the compatible stimuli (big-right) compared to the incompatible stimuli (big-left). As the indicator of interfering effects of the compatible stimuli (big-right) on recall, we predict longer reaction times in distracting conditions (novel and mirrored stimuli). We also predict that the interfering effects in distracting conditions would differ from each other depending on whether the distractors consist of novel stimuli or altered versions of the originally seen stimuli.

## Materials and Methods

### Participants

A total of 100 participants (39 males and 61 females) took part in the experiment. They were university students and staff, aged between 19 and 32 years (*M =* 22.56, *SD* = 2.04). All participants were right-handed according to the Edinburgh Handedness Inventory (Oldfield, 1971; LQ > +50), had normal or corrected-to-normal vision, and no history of neurological or psychiatric disorders. They gave written informed consent in accordance with the ethics committee of the Izmir University of Economics (B.30.2.IEU.0.05.05-020-054), where the study was carried out.

### Stimuli

Thirty-six characters 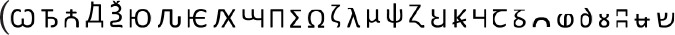 
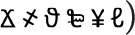
 were obtained from Microsoft Word symbols. Ten were symmetrical (e.g., 
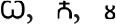
) and 26 were asymmetrical (e.g., 
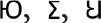
). These characters were assigned to one of three experimental conditions (big-right, big-left, and equal-sized). All but equal-sized condition consisted of symmetrical and asymmetrical characters. In equal-sized condition, we used only asymmetrical characters in order to create proper testing stimuli for the mirrored condition (since the mirrored images of the symmetrical characters would not be proper distractors). Therefore, 12 out of the 26 asymmetrical characters were randomly selected for the equal-sized condition. Then, an equal number of characters were randomly assigned to the big-right and the big-left conditions, chosen from the remaining 24.

These characters were used to construct stimulus pairs. Each pair consisted of two identical characters that varied in size depending on the experimental condition. These were presented on a 19.5-inch LCD display at full 1600 × 900 pixel resolution 40 cm away from the participant, which corresponds to 48°× 32° of visual angle. The pairs of characters were vertically centered and positioned 400 pixels away from each side of the screen. Each character was presented in an imaginary square placeholder. The angular sizes of figures were 8.53°× 8.53° for the larger versions, 2.15°× 2.15° for the smaller versions, and 4.29°× 4.29° for the equal-sized versions. All characters were in black, with a white background.

Three different types of stimulus pairs comprised the experimental conditions. A big-right pair was constructed with a big version of the character on the right, and a small version of the same character on the left. A big-left pair contained a big version on the left and a small version of the same character on the right. For an equal-sized pair, an equal-sized version appeared on each side. Thus, there was a total of 36 pairs (12 big-right pairs, 12 big-left pairs, and 12 equal-sized pairs).

**FIGURE 1:**
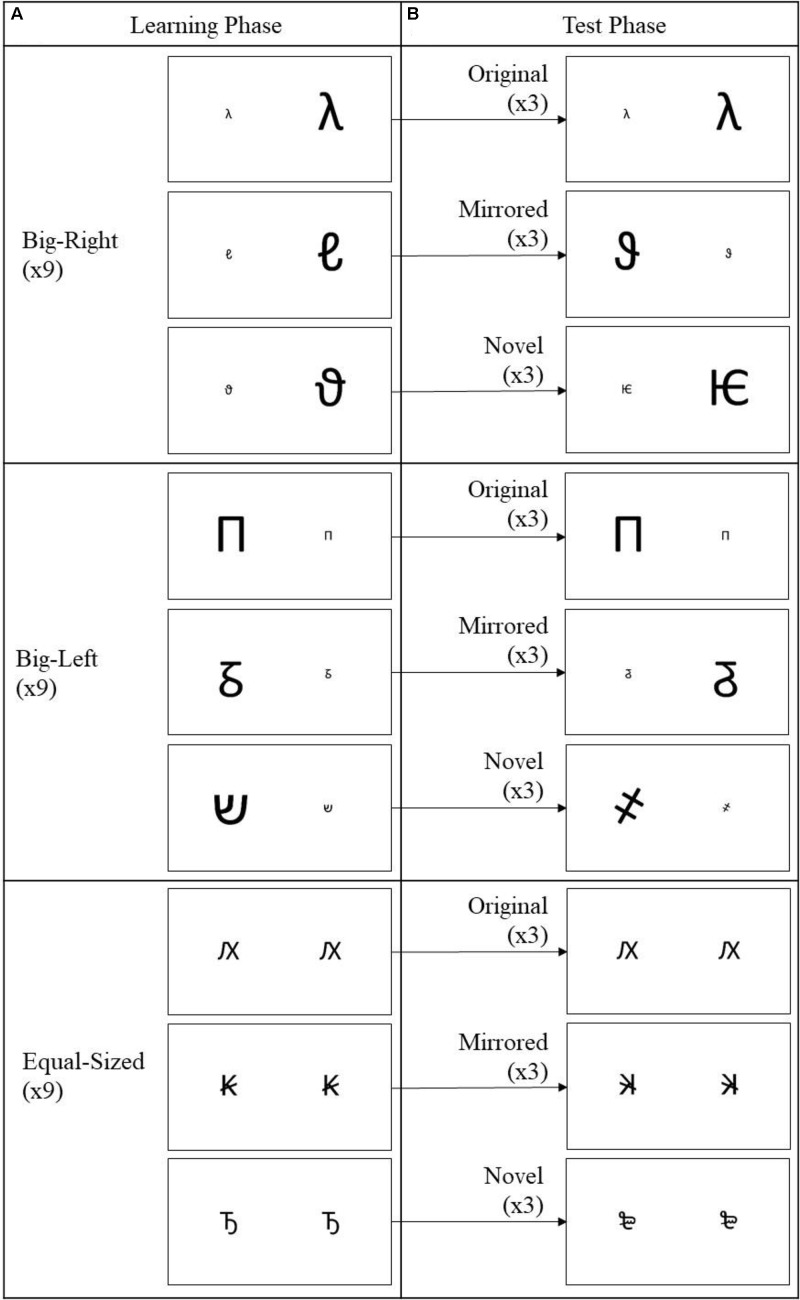
Stimulus pairs used in the learning and test phases of the experiment. In the learning phase **(A)** a total of 27 stimulus pairs with nine stimulus pairs from each experimental condition (big-right, big-left, and equal-sized) were presented in random order. In the test phase **(B)**, nine stimulus pairs (3 × each experimental condition) from the learning phase were used as the original stimuli, nine mirrored version of the stimulus pairs (3 × each experimental condition) from the learning phase, as the mirrored stimuli, and nine new stimulus pairs (3 × each experimental condition), as the novel stimuli.

Nine out of the 12 big-right pairs construed the big-right condition of the learning phase (**Figure 1A**). The remaining three pairs functioned as novel stimuli in the test phase (**Figure 1B**). Three of the nine big-right pairs used in the learning phase functioned as original stimuli in the test phase, and another three different pairs (i.e., not including original stimulus) out of the nine learning pairs functioned as mirrored stimuli in the test phase (**Figures 1A,B**). Mirrored pairs were constructed by horizontally flipping the individual characters and their spatial positions (i.e., left or right). The constructed pairs were randomly assigned to the conditions, and the same procedure was followed for big-left and equal-sized pairs.

### Procedure

**FIGURE 2:**
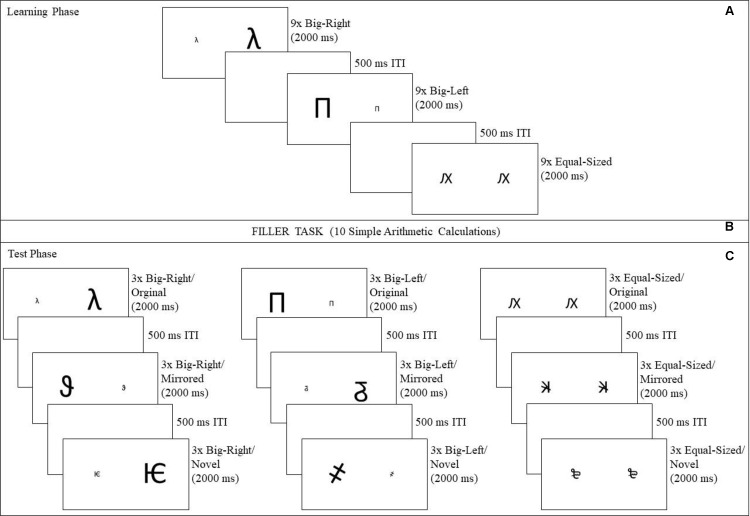
Experimental procedure followed in the study. The experiment consisted of two phases, a learning phase and a test phase. In the learning phase **(A)**, a total of 27 stimuli were presented for 2,000 ms with 500 ms inter-stimulus intervals. In order to prevent the participants from rehearsing after the training phase, a filler task was used **(B)**, requiring participants to perform a total of 10 simple arithmetic calculations. In the test phase **(C)**, participants indicated whether or not they considered that had seen the stimuli during the learning phase by pressing B key for “YES” response or N key for “NO” response as quickly as possible, yielding accuracy and reaction time measures.

The participants were seated comfortably in a dimly lit sound-attenuating chamber, and were instructed to remain in the same position throughout the experiment. The experiment was carried out in two successive phases, with a filler task between (**Figure 2**). In the learning phase (**Figure 2A**), the participants were presented a total of 27 stimulus pairs, which consisted of nine pairs from each experimental condition (big-right, big-left, and equal-sized). They were instructed to memorize as many pairs as possible, by considering the form, size, and spatial location of the stimuli on the screen. At the end of the learning phase, a brief filler task was introduced to prevent any rehearsal (**Figure 2B**). The filler task consisted of 10 simple arithmetic calculations^1^ [e.g., (76 ÷ 2) × 4 and (979 - 779) ÷ 2].

A total of 27 stimulus pairs were presented in the test phase (**Figure 2C**): nine in the original stimulus category consisting of an equal number of big-right, big-left, and equal-sized pairs; nine in the mirrored stimulus category consisting of an equal numbers of big-right, big-left, and equal-sized pairs; and nine in novel stimulus category consisting of an equal numbers of big-right, big-left, and equal-sized pairs. Each pair was presented in a randomized order for 2,000 ms, with a 500 ms inter-stimulus interval both in the learning and test phases. The participants were asked to indicate whether they had previously seen that specific pair of characters in the learning phase by pressing B key for a “YES” response or N key for a “NO” response as quickly as possible. The participants used their right index finger for the “YES” response and their right middle finger for the “NO” response. Their responses yielded two measures, accuracy of recall and reaction times. SuperLab 4.5^2^ (Cedrus Corporation, United States) was used to control stimulus presentations and response recordings during the experimental sessions. It took about 10 min for each participant to complete the task.

### Data Analysis

Experimental condition (big-right, big-left, and equal-sized) and stimulus category (original, mirrored, and novel) were within-participant variables. Accuracy of recall and reaction time were recorded as dependent measures. Response accuracy was examined within the framework of signal detection theory. In addition, for each condition, mean reaction time scores were calculated disregarding the accuracy of responses^3^. A 3 (experimental condition) × 3 (stimulus category) repeated analysis of variance (ANOVA) was conducted to analyze the reaction time data. In the analysis of reaction time data, in all planned contrasts, the original stimulus category was used as the reference condition for the stimulus category, and the big-right condition was used as the reference condition for the experimental condition.

#### Signal Detection Analysis

Signal detection theory is an accepted procedure when signal and noise trails must be discriminated (Stanislaw and Todorov, 1999). In this study, we define signal trials as those that contain previously studied stimuli, and noise trials as those that contain distractor stimuli of yes/no task (e.g., seen/unseen). On signal trials, “yes” responses are correct and are named as *hit*. On noise trials, however, “yes” responses are incorrect and are termed as *false alarm*. The hit rate (the probability of responding yes on signal trials) and the false alarm rate (the probability of responding yes on noise trials) are the indicators of performance in a yes/no task. The hit rate is calculated by dividing the number of hits by the total number of signal trials. The false alarm rate is calculated by dividing the number of false alarms by the total number of noise trials. Based on these hit and false-alarm rates, two signal detection parameters are calculated: sensitivity (*d′)* and response bias (*c*).

*d′* represents the participants’ ability to discriminate the “signals” (hits) from the “noise” (false alarms) (Wilson and Swets, 1954). This is calculated by subtracting the *z*-score of false-alarm rate from the *z*-score of hit rate. A *d′* value of 0 (zero) indicates an inability to distinguish signal from noise, whereas higher values reflect more “yes” responses to previously studied stimuli, and more “no” responses to distracting stimuli (Lockhart and Murdock, 1970). *c* is calculated by averaging the *z* scores of hit and false alarm rates, then multiplying the result by -1. Negative values of *c* indicate a bias toward “yes” responses, and positive values, in favor of “no” responses (Stanislaw and Todorov, 1999).

Accordingly, in the present study, stimulus pairs of the original stimulus category were identified as the signal, and stimulus pairs of novel and mirrored stimulus categories, as the noise. Thus, “yes” responses in the original stimulus category constituted hits, and “yes” responses in the novel and mirrored stimulus categories, false alarms. In regard to the experimental conditions, hit and false alarm values were calculated in six parts (**Table 1**). *d′* and *c* parameters for each participant were calculated based on these parts. For example, to calculate *d′* and *c* values in the original versus novel comparison of the big-right condition (see row 1/**Table 1**), signal trials were acquired from the big-right/original stimuli, and hits were gathered from “yes” responses to those stimuli. For the noise trials, big-right/novel stimuli were used, and false alarms were obtained from the “yes” responses to those stimuli.

**Table 1:** Stimulus category comparisons by experimental conditions used for calculating hit and false alarm rates.

		Previously	Distractor
		studied (hits)	(false alarms)
Original versus novel	1	Big-right/original	Big-right/novel
	2	Big-left/original	Big-left/novel
	3	Equal-sized/original	Equal-sized/novel
Original versus mirrored	4	Big-right/original	Big-right/mirrored
	5	Big-left/original	Big-left/mirrored
	6	Equal-sized/original	Equal-sized/mirrored


## Results

### Accuracy of Recall

Four separate one-way repeated ANOVAs were performed both in the original versus mirrored comparison and the original versus novel comparison for *d′* and *c* parameters. In the analysis of the signal detection parameters, the big-right condition was used as the reference condition in all planned contrasts. **Table 2** shows mean and standard deviation values of *d′* and *c* parameters in the original versus novel, and the original versus mirrored comparisons by experimental conditions.

**Table 2:** Mean and standard deviation values of *d′* and *c* parameters in the original versus novel and the original versus mirrored comparisons by experimental conditions.

	Experimental condition					
	Big-right		Big-left		Equal-sized	
	*d′*	*c*	*d′*	*c*	*d′*	*c*
Original	1.229	-0.145	1.415	0.279	1.546	0.242
versus novel	(0.861)	(0.407)	(0.638)	(0.381)	(0.687)	(0.363)
Original	0.440	-0.538	0.293	-0.272	0.528	-0.283
versus mirrored	(0.771)	(0.498)	(0.859)	(0.497)	(0.794)	(0.500)


**FIGURE 3:**
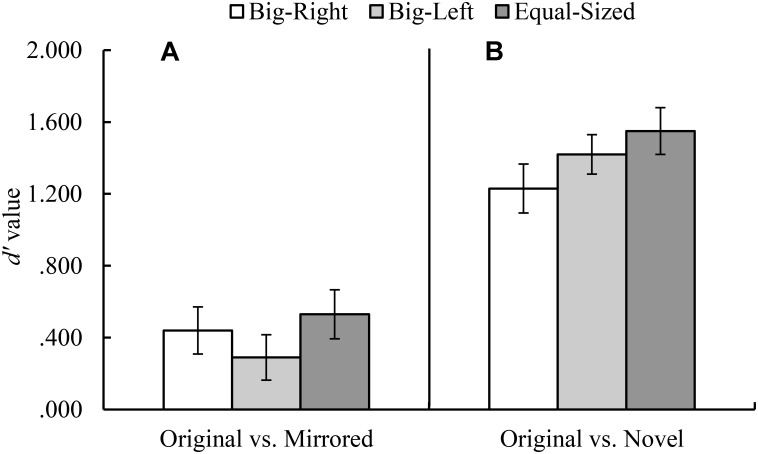
Mean *d′* values for the original versus mirrored **(A)**, and the original versus novel **(B)** stimulus category comparisons by experimental condition (Error bars represent 95% CI adjusted for repeated measures).

**FIGURE 4:**
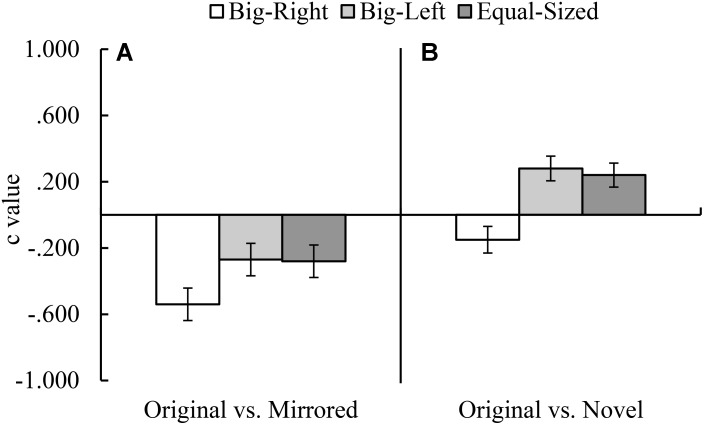
Mean *c* values for the original versus mirrored **(A)**, and the original versus novel **(B)** stimulus category comparisons by experimental condition (Error bars represent 95% CI adjusted for repeated measures).

In the original versus novel stimulus category comparison for *d′* parameter, Mauchly’s test indicated that assumption of sphericity had been violated, χ^2^_(2)_ = 10.59, *p* = 0.005. Therefore, degrees of freedom were corrected by using Greenhouse–Geisser estimates of sphericity. The results of the analysis indicated a significant experimental condition effect for *d′* parameter, *F*_(1.81,177.62)_ = 5.23, *p* = 0.008, η^2^ = 0.05 (**Figure 3B**). Contrasts based on *d′* values indicated that the participants in the big-right condition performed worse than those in the equal-sized condition, *F*_(1,98)_ = 8.08, *p* = 0.005, η^2^ = 0.08, in discriminating the signal from noise; but they performed similarly in the big-left condition, *F*_(1,98)_ = 3.59, *p* = 0.061. In the original versus novel stimulus category comparison for *c* parameter, it was found a significant effect of experimental condition, *F*_(2,194)_ = 49.46, *p* < 0.001, η^2^ = 0.34 (**Figure 4B**). Contrasts analysis based on *c* values revealed that the participants significantly favored the “yes” response in the big-right condition compared to the big-left condition, *F*_(1,97)_ = 81.33, *p* < 0.001, η^2^ = 0.47, and the equal-sized condition, *F*_(1,97)_ = 67.04, *p* < 0.001, η^2^ = 0.41.

In the original versus mirrored stimulus category comparison, *d′* values did not differ across the experimental conditions, *F*_(2,198)_ = 2.53, *p* = 0.082 (**Figure 3A**); on the other hand, *c* values indicated a significant effect of the experimental condition, *F*_(2,198)_ = 11.65, *p* < 0.001, η^2^ = 0.11 (**Figure 4A**). Planned contrasts based on the *c* values indicated that the participants favored the “yes” response more in the big-right condition compared to both the big-left, *F*_(1,99)_ = 20.42, *p* < 0.001, η^2^ = 0.17, and equal-sized conditions, *F*_(1,99)_ = 15.36, *p* < 0.001, η^2^ = 0.13.

### Reaction Time

The mean and standard deviation values of reaction time scores for the experimental conditions by stimulus categories are shown in **Table 3**. Mauchly’s test indicated that assumption of sphericity had been violated for the main effect of experimental condition, χ^2^_(2)_ = 12.37, *p* = 0.002 and for the interaction between experimental condition and stimulus category, χ^2^_(9)_ = 22.71, *p* = 0.007. Therefore, degrees of freedom were corrected by using Greenhouse–Geisser estimates of sphericity. A 3 × 3 ANOVA for repeated measures indicated a significant main effect of the experimental condition on reaction time, *F*_(1.79,177.02)_ = 22.59, *p* < 0.001, η^2^ = 0.19. Planned contrasts revealed that the reaction time for the big-right (*mean* = 1483.66, *SE* = 44.36) condition was significantly longer than the big-left (*mean* = 1356.03, *SE* = 32.35), *F*_(1,99)_ = 15.79, *p* < 0.001, η^2^ = 0.14, and equal-sized (*mean* = 1270.76, *SE* = 25.80) conditions, *F*_(1,99)_ = 35.32, *p* < 0.001, η^2^ = 0.26. There was also a significant main effect of the stimulus category on reaction time, *F*_(2,198)_ = 25.71, *p* < 0.001, η^2^ = 0.21. The contrasts analysis indicated that the mean reaction time for the original stimulus category (*mean* = 1317.98, *SE* = 30.36) was significantly shorter than that for the mirrored stimulus category (*mean* = 1513.68, *SE* = 42.21), *F*_(1,99)_ = 36.17, *p* < 0.001, η^2^ = 0.27; however, it did not differ from the mean reaction time for the novel stimulus category (*mean* = 1288.80, *SE* = 31.93), *F*_(1,99)_ = 0.78, *p* = 0.380.

**Table 3:** Mean and standard deviation values of reaction time scores in experimental conditions by stimulus categories.

		Experimental condition		
		Big-right	Big-left	Equal-sized
Stimulus category	Original	1262.105	1431.962	1259.870
		(387.928)	(465.016)	(323.795)
	Novel	1538.702	1158.742	1168.950
		(594.657)	(316.959)	(327.136)
	Mirrored	1680.182	1477.397	1383.448
		(701.887)	(508.622)	(427.413)


**FIGURE 5:**
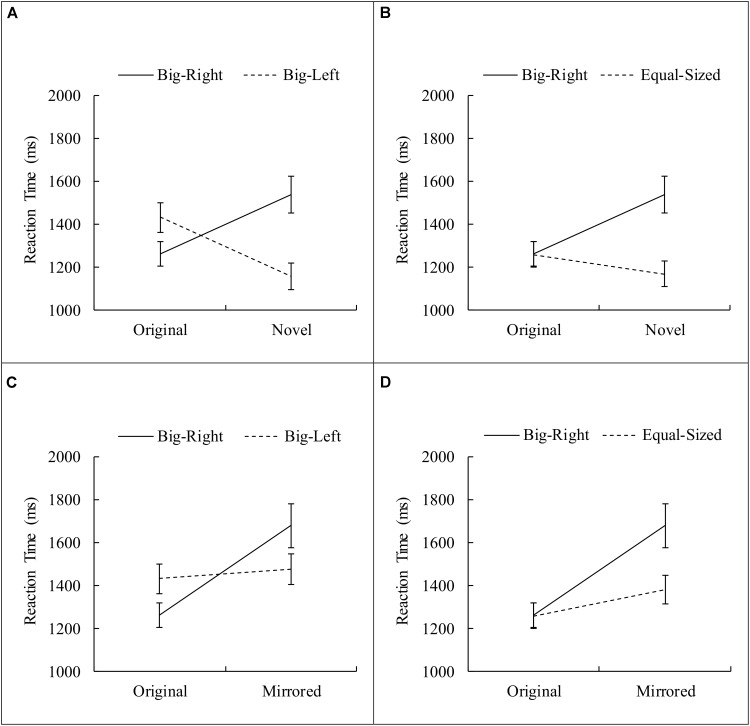
Planned contrasts for the interaction between experimental condition and stimulus category. **(A)** Shows data for original vs. novel and big-right vs. big-left; **(B)** shows data for original vs. novel and big-right vs. equal-sized; **(C)** shows data for original vs. mirrored and big-right vs. big-left; **(D)** shows data for original vs. mirrored and big-right vs. equal-sized (Error bars represent 95% CI adjusted for repeated measures).

There was a significant interaction effect between the experimental condition and the stimulus category, *F*_(3.61,356.96)_ = 16.21, *p* < 0.001, η^2^ = 0.14. Four planned contrasts were performed comparing each level of stimulus categories (i.e., novel and mirrored) to the original stimulus category across each level of experimental conditions (i.e., big-left and equal-sized) comparing to the big-right condition. The first contrast that compared the original stimulus category to the novel stimulus category in respect to the big-right and big-left conditions was significant, *F*_(1,99)_ = 50.58, *p* < 0.001, η^2^ = 0.34. This significant interaction indicated that the participants responded faster to the big-right than to the big-left stimuli in the original stimulus category; however, in the novel stimulus category, reaction times were slower on the big-right compared to the big-left condition (**Figure 5A**). The second contrast was performed to compare the reaction time data obtained from the original stimulus category and from the novel stimulus category, in respect to the big-right and equal-sized conditions. This interaction was also significant, *F*_(1,99)_ = 30.44, *p* < 0.001, η^2^ = 0.24, suggesting that reaction times were similar for both the big-right and equal-sized conditions in the original stimulus category; they were slower for the big-right condition than the equal-sized condition in the novel stimulus category (**Figure 5B**). The third contrast which compared the original and mirrored stimulus categories in respect to the big-right and big-left conditions was significant, *F*_(1,99)_ = 24.79, *p* < 0.001, η^2^ = 0.20. This significant interaction suggested that participants responded faster to the big-right stimuli than to the big-left stimuli in the original stimulus category; but in the mirrored stimulus category, they responded more slowly to the big-right stimuli than to the big-left stimuli (**Figure 5C**). The final contrast, which compared the original and mirrored stimulus categories in respect to the big-right and equal-sized conditions, was also significant, *F*_(1,99)_ = 14.31, *p* < 0.001, η^2^ = 0.13. This significant interaction implied that the reaction times obtained from the big-right and equal-sized conditions on the original stimulus category were similar; however, they were longer on the big-right condition than the equal-sized condition in the mirrored stimulus category (**Figure 5D**).

## Discussion

The aim of the present study was to see whether we could elicit any evidence for an association between size and space by investigating the recognition memory. Particularly, we predicted that compatibility between physical size – spatial position of stimulus and its memory representation would affect the sensitivity, response bias, and reaction time for recognition. Hence, the big-right (also, small-left) stimuli appeared to be associated significantly faster responses when tested with original pairs of stimuli. Whereas, big-right distractors (i.e., novel and mirrored trials) produced increased number of false “yes” responses and longer reaction time measures. This implies a compatibility effect between size and space regarding memory representations of size and horizontal position of stimuli. Thus, an important implication of this finding pertains to ATOM (“A Theory Of Magnitude”; Walsh, 2003, 2015; Bueti and Walsh, 2009) which predicts the existence of a generalized, integrating magnitude-processing system that helps the control of complex actions by providing a ground for interacting of magnitudes such as number, space, and time.

We suggest from our results that the system might be activated upon the detection of a size difference between the two characters. Compared to the other experimental conditions, the equal-sized condition yielded better discrimination, lower response bias, and similar reaction times regardless of the stimulus category tested with (whether or not the test stimulus was original, mirrored, or novel). The equal-sized condition probably would not activate the magnitude comparing process, because it would have been redundant, presumably the ATOM operates on the size–space compatibility. Similarly, when there was size difference in a pair of characters (i.e., big-left and big-right conditions), we observed differences in reaction time and the signal detection parameters. Therefore, we conceived that this generalized magnitude processing system might be sensitive to the size differences for its activation: An internal on-off switch might be operated by a perceptual process upon the detection of a size difference.

However, ATOM does not assume the direction of the size-spatial position association readily (Walsh, 2003, 2015; Bueti and Walsh, 2009). Then, this left-to-right oriented size–space compatibility effect we observed, calls for an explanation. Several accounts may be offered. Some recent data has indicated functional differences between the two hands. The right hand generally is the dominant hand and it is stronger than the left hand (Hepping et al., 2015). Hence, perhaps the manual-motor dominancy gives way to the faster right-hand responses to the larger magnitudes (Incel et al., 2002). In our study, our participants were all right handers, and they used only their right hand to respond to the stimuli. Moreover, they responded to the stimuli by hitting the “b” or “n” keys which are located centrally on a QWERTY keyboard. Therefore, we do not consider this as a valid account for our experimental setup. On the other hand, if the compatibility between physical size and space utilizes the same sources as the SNARC, then we may explain the direction of the effect based on the converging support of acculturation such as diffusion of spatial-directional scanning habits from reading into the domain of numerical (Dehaene et al., 1993) or other magnitude-related cognition, consensually developed action patterns (Lindemann et al., 2011), and the influence of external representations such as graphs and notation systems (Bender et al., 2010; Tversky, 2011).

Alternatively, given the methodological differences between our study and the previous work, we offered an account based on congruency between the short-term and long-term associations. This account adapted from dual-route models (Tagliabue et al., 2000) by Wühr and Seegelke (2018) to explain how relevant and irrelevant stimulus features evoke short-term and long-term associations. The account predicts that when there is congruency between these two associations, both processing routes activate the correct response resulting shorter reaction times and better accuracy. Whereas in incongruent conditions, the long-term association would interfere with selection of the correct response resulting longer reaction times and lessened accuracy. Similarly, we assume that the information about the spatial orientation of magnitudes has already been stored in memory of long term association between size and space, probably through acculturation. In fact, according to the instances theory (Choplin and Logan, 2005), our past experiences are the main source for the magnitude–space associations, thus people rely on the instances available from long-term memory (Logan, 1988). Likewise, this congruency account could be applied to size–space compatibility effect that we observed in our study.

We conceived that short-term associations reflect the decisions of participants about the test stimuli based on acquired size – spatial position information through the learning phase of the study. The level of the short-term association therefore depends, in part, on the compatibility between the test stimuli and the stimuli studied in learning phase. Whereas, the long-term associations refer to the information about the size – spatial position had already been acquired. The level of the long-term association depends on the compatibility between the test stimuli and the available information acquired through long-term processing. In the original stimulus category, the test stimuli were the same as the stimuli presented during the learning phase. Here, participants showed shorter reaction times to the big-right stimuli, compared to the big-left stimuli. Given the fact that both the big-right and big-left stimuli were tested by their exact copies (short-term compatibility; see **Table 4**, rows 1 and 2), the difference observed in reaction times may be attributed to the differential long-term compatibility levels of the big-right (long-term compatibility; see **Table 4**, row 1) and big-left (long-term incompatibility; see **Table 4**, row 2) stimuli. Hence, the congruency between short- and long-term associations in the big-right condition resulted in decreased reaction times.

**Table 4:** Congruency between short- and long-term associations in big-bight and big-left conditions by different stimulus categories.

	Experimental condition	Stimulus category	Short-term	Long-term	Congruency
1	Big-right	Original	Compatible	Compatible	Congruent
2	Big-left	Original	Compatible	Incompatible	Incongruent
3	Big-right	Novel	Incompatible	Compatible	Incongruent
4	Big-left	Novel	Incompatible	Incompatible	Congruent
5	Big-right	Mirrored	Incompatible	Incompatible	Congruent
6	Big-left	Mirrored	Incompatible	Compatible	Incongruent


This congruency effect was also evident in the novel condition in which participants were tested with stimuli that they did not studied in learning phase. Interestingly, this time the culprit was the big-left stimuli: Being as the novel stimuli, both the big-right and big-left stimuli were not encoded in the learning phase (short-term incompatibility; **Table 4**, rows 3 and 4); however, the big-left stimuli were structurally incompatible with the long-term association code (long-term incompatibility; **Table 4**, row 4). This reflects a negative congruency between the short- and long-term associations in the big-left condition. Hence, we obtained shorter reaction times and lower false alarm rates. On the other hand, in the big-right stimulus category, the test stimuli were compatible with the long-term association code (long-term compatibility; **Table 4**, row 3). This reflects an incongruency between the short- and long-term associations in the big-right condition. As a result, we observed an interference on decisions of participants presumably originated from the long-term association code. This interference was evidenced by longer reaction times and higher false alarm rates. This indicates a long-term interference effect on congruency between the short- and long-term associations.

Finally, in the mirrored stimulus category, participants were tested with mirrored (horizontally flipped) versions of the stimuli. Being as the mirrored stimuli, both the big-right and big-left stimuli were not the same as what had been seen in learning phase (short-term incompatibility; **Table 4**, rows 5 and 6). However, the big-right mirrored stimuli were structurally incompatible with the long-term association code (long-term incompatibility; **Table 4**, row 5). Hence, we observed another negative congruency effect in the mirrored condition with the big-right stimuli. However, this time, the observed congruency resulted in increased reaction times and increased response bias in favor of “yes.” This is an unexpected finding, because instead of the expected facilitating effect of the congruency, we obtained an interference. Obviously, in the big-right mirrored trials, the long-term compatible big-right stimuli seen in the learning phase were tested with the long-term incompatible stimuli. We speculate, therefore, that the compatible stimuli in the learning phase might be responsible for the interference, reflecting a possible short-term interference effect on congruency between the short- and long-term associations. Clearly, future research is required to test this speculative position.

## Conclusion

To conclude, we provided evidence for the relationship between stimulus physical size and how they processed spatially by employing a false memory procedure. To the best of our knowledge, this is the first work that uses memory errors to investigate the size–space relationship. Also, this piece of evidence supported the existence of a generalized magnitude processing system assumed by ATOM. Since the ATOM lacks an account of the direction of the size – spatial position association, we offered an interplay between the short-term and long-term associations which determines the direction of the spatial organization of physical magnitudes. Thus, in line with Ginsburg and Gevers (2015) and Gut and Staniszewski (2016), spatial response biases might result from the activation of both pre-existing positions and from temporary space associations at the same time. Finally, we offer a size–space compatibility account based on the congruency between the short- and long-term associations which produce local compatibilities. We think that our study takes place in the intersection of shared-representation and shared-decision accounts and offers more eclectic approach toward the understanding of magnitude–space association. Future research is required to further test the suggestive evidence provided by the present study.

## Author Contributions

All authors listed have made a substantial, direct and intellectual contribution to the work, and approved it for publication.

## Conflict of Interest Statement

The authors declare that the research was conducted in the absence of any commercial or financial relationships that could be construed as a potential conflict of interest.

## Footnotes

1 Arithmetic problems are commonly presented as filler task in false memory studies (e.g., Coane and McBride, 2006). In order to ensure completeness, we checked the answers for their accuracy, and found that the arithmetic problems were 85–90% percent solved with accuracy.

2 http://www.superlab.com/

3 In analyses of repeated measures, when we obtain a “0” accuracy score from a participant in a specific condition (e.g., mirrored stimulus category), it is not possible to use other responses of the same participant to calculate the model parameters. Further, when the reaction time data for only correct responses were analyzed, the results maintained for the original main and interaction effects (see the **Supplementary Material**). Thus, the reaction time data for both correct and incorrect responses were reported.
