# Supplementary material for: Compatibility Between Physical Stimulus Size – Spatial Position and False Recognitions
Source: Front Psychol. 2018 Aug 14;9:1457. doi: 10.3389/fpsyg.2018.01457 (PMC6102480; doi:10.3389/fpsyg.2018.01457)
Supplement: Supplementary file 1 [file Table_1.DOCX]

Supplementary Material

Compatibility between Physical Stimulus Size – Spatial Position and False Recognitions

Seda Dural^1*^, Birce Begüm Burhanoğlu, Nilsu Ekinci, Emre Gürbüz, İdil Umay Akın, Seda Can, Hakan Çetinkaya

*** Correspondence:** Seda Dural: [seda.dural@ieu.edu.tr](mailto:seda.dural@ieu.edu.tr)

# Reaction Time Results only for Correct Responses

A 3 x 3 ANOVA for repeated measures indicated a significant main effect of the experimental condition on reaction time, *F*_(2, 68)_ = 5.60, *p* = .006, *η*² = .14. Planned contrasts revealed that the reaction time for the big-right condition was significantly longer than the equal-sized condition, *F*_(1, 34)_ = 9.82, *p* = .004, *η*² = .22; however it did not differ from the big-left condition, *F*_(1, 34)_ = .64, *p* = .428. There was also a significant main effect of the stimulus category on reaction time, *F*_(1.57, 53.45)_ = 25.37, *p* < .001, *η*² = .43. The contrasts analysis indicated that the mean reaction time for the original stimulus category was significantly shorter than that for the mirrored stimulus category, *F*_(1, 34)_ = 20.73, *p* < .001, *η*² = .38; however it did not differ from the mean reaction time for the novel stimulus category, *F*_(1, 34)_ = 3.33, *p* = .077.

There was a significant interaction effect between the experimental condition and the stimulus category, *F*_(2.54, 86.43)_ = 2.87, *p* = .050, *η*² = .09. Four planned contrasts were performed comparing each level of stimulus categories (i.e. novel and mirrored) to the original stimulus category across each level of experimental conditions (i.e. big-left and equal-sized) comparing to the big-right condition. The first contrast that compared the original stimulus category to the novel stimulus category in respect to the big-right and big-left conditions was significant, *F*_(1, 34)_ = 4.72, *p* = .037, *η*² = .12. This significant interaction indicated that reaction times were similar for both the big-right and big-left conditions in the original stimulus category; they were slower for the big-right condition than the big-left condition in the novel stimulus category. The second contrast was performed to compare the reaction time data obtained from the original stimulus category and from the novel stimulus category, in respect to the big-right and equal-sized conditions and this interaction was not significant, *F*_(1, 34)_ = .77, *p* = .388. The third contrast which compared the original and mirrored stimulus categories in respect to the big-right and big-left conditions was not significant, *F*_(1, 34)_ = .07, *p* = .794. The final contrast, which compared the original and mirrored stimulus categories in respect to the big-right and equal-sized conditions, was significant, *F*_(1, 34)_ = 5.15, *p* = .030, *η*² = .13. This significant interaction implied that the reaction times obtained from the big-right and equal-sized conditions on the original stimulus category were similar; however, they were longer on the big-right condition than the equal-sized condition in the mirrored stimulus category.
